# Serum cold-inducible RNA-binding protein levels as a potential biomarker for systemic sclerosis-associated interstitial lung disease

**DOI:** 10.1038/s41598-023-32231-1

**Published:** 2023-03-28

**Authors:** Issei Omori, Hayakazu Sumida, Ayaka Sugimori, Moe Sakakibara, Mariko Urano-Takaoka, Okuto Iwasawa, Hinako Saito, Ai Matsuno, Shinichi Sato

**Affiliations:** 1 Department of Dermatology, Faculty of Medicine, The University of Tokyo, 7-3-1, Hongo, Bunkyo-ku, Tokyo, 113-8655 Japan; 2 Scleroderma Center, The University of Tokyo Hospital, Bunkyo-Ku, Tokyo, Japan

**Keywords:** Autoimmunity, Autoimmune diseases

## Abstract

Systemic sclerosis (SSc) is a complex autoimmune disease characterized by fibrotic, inflammatory, and vascular dysfunction. Danger-associated molecular patterns (DAMPs)-mediated inflammasome activation has been reported to be involved in the pathogenesis of SSc. Cold-inducible RNA-binding protein (CIRP) is newly identified as a DAMP. Here we examined the clinical significance of serum levels of CIRP in 60 patients with SSc and 20 healthy control patients (HCs) using an enzyme-linked immunosorbent assay. Serum CIRP levels in diffuse cutaneous SSc (dcSSc) patients were significantly increased compared with limited cutaneous SSc (lcSSc) patients or HCs. When examining the relationship with SSc-specific parameters, serum CIRP levels with the presence of interstitial lung disease (ILD) were higher than those without ILD. In detail, serum CIRP levels correlated negatively with the percent predicted diffusing capacity for carbon monoxide and positively with levels of Krebs von den Lungen-6. In addition, elevated serum CIRP levels declined along with decreased SSc-ILD activity in patients who received immunosuppressive therapy. These results suggest that CIRP may play a role in the development of ILD in SSc. Moreover, CIRP could serve as a useful serological marker of SSc-ILD in terms of disease activity and therapeutic effects.

## Introduction

Systemic sclerosis (SSc) is a multisystem autoimmune and vascular disorder of unknown etiology resulting in excessive fibrosis of the skin, lung, and other internal organs^1^. Interstitial lung disease (ILD) is a common manifestation of SSc and a leading cause of death^2^. SSc-associated ILD (SSc-ILD) runs a highly variable course and several promising serum biomarkers of SSc-ILD have been suggested. However, few of them could help estimate the progression of SSc-ILD. Therefore, new prediction tools are highly desired^3^.

Recently, danger-associated molecular patterns (DAMPs)-mediated inflammasome has drawn much attention as a part of disease-associated molecules in SSc^4, 5^ and idiopathic pulmonary fibrosis^6^. Cold-inducible RNA-binding protein (CIRP) has been recently identified as a DAMP^7^ and is a highly conserved 172-amino acid nuclear protein that belongs to the family of cold shock proteins^8, 9^. CIRP is a general stress-response protein upregulated by hypoxia, UV radiation, glucose deprivation, heat stress, and H_2_O_2_^10^. CIRP is ubiquitously expressed in various tissues, including the skin, lungs, and heart^11^. Recent studies have discovered that CIRP is endowed with extracellular signaling functions on various cell types^9^. CIRP is released from host cells to the extracellular space acting as a DAMP^7^. Recently, CIRP has been reported to be involved in the pathogenesis of idiopathic pulmonary fibrosis^12^. However, the involvement of CIRP in SSc is not well known. This study measured serum CIRP levels in 60 SSc patients to investigate associations with clinical symptoms, complications, and other biomarkers. Further analyzes were also planned to explore the potential of CIRP as a predictive marker of disease activity or therapeutic effect.

## Results

### Elevated serum CIRP levels in SSc

**Figure 1:**
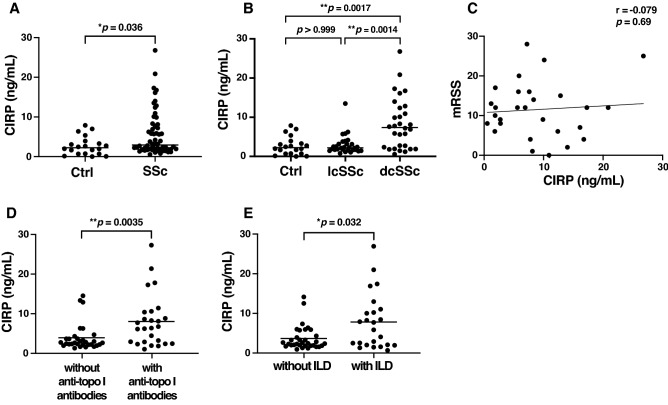
Elevated serum CIRP levels in diffuse cutaneous SSc patients associated with anti-topoisomerase I antibodies and interstitial lung disease. (**A**) Serum cold-inducible RNA-binding protein (CIRP) levels in systemic sclerosis (SSc; *n* = 60) and healthy controls (Ctrl; *n* = 20). Serum CIRP levels were determined by a specific enzyme-linked immunosorbent assay. (**B**) Serum CIRP levels in diffuse cutaneous SSc (dcSSc; *n* = 30), limited cutaneous SSc (lcSSc; *n* = 30), and healthy controls (Ctrl; *n* = 20). (**C**) Correlation analysis of serum CIRP levels and modified Rodnan total skin thickness score (mRSS) in SSc patients. The solid line represents the regression line. (**D**) Serum CIRP levels in SSc patients with anti-topoisomerase I (topo I) antibodies and those without anti-topo I antibodies. (**E**) Serum CIRP levels in SSc patients with interstitial lung disease (ILD) and those without ILD. *p < 0.05, **p < 0.01.

Serum CIRP levels in SSc patients were significantly higher than those in healthy controls (median [25–75th percentiles], 2.94 [1.85–7.64] vs. 2.23 ng/mL [0.73–3.45], *p* = 0.036; Fig. 1A). For the SSc subgroups, serum CIRP levels in diffuse cutaneous SSc (dcSSc) patients were significantly increased compared with limited cutaneous SSc (lcSSc) patients (7.40 [2.45–12.01] vs. 2.19 ng/mL [1.67–3.17], *p* = 0.0014; Fig. 1B). Meanwhile, there were no significant differences in the levels of CIRP between lcSSc and healthy controls (2.19 [1.67–3.17] vs. 2.23 ng/mL [0.73–3.45], *p* > 0.999; Fig. 1B).

### Clinical association of serum CIRP levels in SSc

Serum CIRP levels showed no correlation with skin scores (*r* = − 0.079, *p* = 0.69; Fig. 1C). Considering the prevalence of cutaneous vascular symptoms, we focused on Raynaud’s phenomenon, nail fold bleeding, telangiectasia, pitting scars, and digital ulcers. However, the presence of each cutaneous vascular symptom did not affect serum CIRP levels (Table 1). Regarding visceral involvement, the presence of esophageal dysfunction, heart involvement, scleroderma renal crisis, and elevated right ventricular systolic pressure did not show significant differences in terms of serum CIRP levels (Table 1). There was no association between serum CIRP levels and the presence of anti-centromere antibodies (2.14 [1.62–2.82] vs. 2.23 ng/mL [0.73–3.45], *p* > 0.999) or anti-RNA polymerase III antibodies (2.24 [1.59–3.51] vs. 2.23 ng/mL [0.73–3.45], *p* > 0.999), while serum CIRP levels with the presence of anti-topoisomerase I (topo I) antibodies were higher than those without anti-topo I antibodies (6.05 [2.36–9.50] vs. 2.14 ng/mL [1.69–3.17], *p* = 0.0035; Fig. 1D). Additionally, serum CIRP levels of SSc patients with ILD were higher than those without ILD (6.65 [1.92–10.30] vs. 2.32 ng/mL [1.72–4.18], *p* = 0.032; Fig. 1E).

**Table 1:** Association of serum CIRP levels with clinical features in SSc patients.

Characteristic	Serum CIRP levels (ng/ml)		*p* values
	Patients with symptoms	Patients without symptoms	
Cutaneous vascular symptoms			
Raynaud’s phenomenon	2.72 [1.78–7.59] (53)	3.93 [3.03–4.85] (3)	0.79
Nail fold bleeding	2.62 [1.83–5.80] (36)	3.67 [1.81–10.30] (20)	0.48
Telangiectasia	2.76 [1.45–12.38] (9)	5.71 [1.91–8.22] (31)	0.59
Pitting scars	2.13 [1.45–6.84] (12)	3.86 [1.99–7.73] (38)	0.16
Digital ulcers	2.35 [1.81–6.24] (9)	3.68 [1.93–9.50] (34)	0.54
Organ involvement			
Interstitial lung disease	6.65 [1.92–10.30] (24)	2.32 [1.72–4.18] (33)	0.032
Esophageal dysfunction	3.18 [1.88–7.95] (39)	2.14 [1.68–3.64] (14)	0.16
Heart involvement	6.15 [5.17–7.14] (2)	3.16 [1.88–7.68] (47)	
Scleroderma renal crisis	5.77 [5.77- 5.77] (1)	3.14 [1.87–8.04] (46)	
Elevated RVSP	5.80 [3.51–7.15] (6)	2.50 [1.81–7.20] (49)	0.51

Clinical manifestations are defined as follows: pitting scars, pinhole-sized digital hyperkeratosis; digital ulcers, a loss of epithelialization; intestinal lung disease, the presence of ground-glass opacity and/or reticular pattern on computed tomography analysis; esophageal dysfunction, abnormal reflux of gastric contents or symptoms associated with gastroesophageal reflux disease; heart involvement, symptomatic pericarditis, clinical evidence of left ventricular congestive heart failure, and/or arrhythmias requiring treatment; elevated right ventricular systolic pressure (RVSP), ≥ 35 mmHg on echocardiogram; scleroderma renal crisis, malignant hypertension and/or rapidly progressive renal failure, malignant hypertension and/or rapidly progressive renal failure. Values represent the median with 25–75 percentiles in square brackets and patient numbers in parentheses.

### Correlation between serum CIRP levels and SSc-ILD parameters

**Figure 2:**
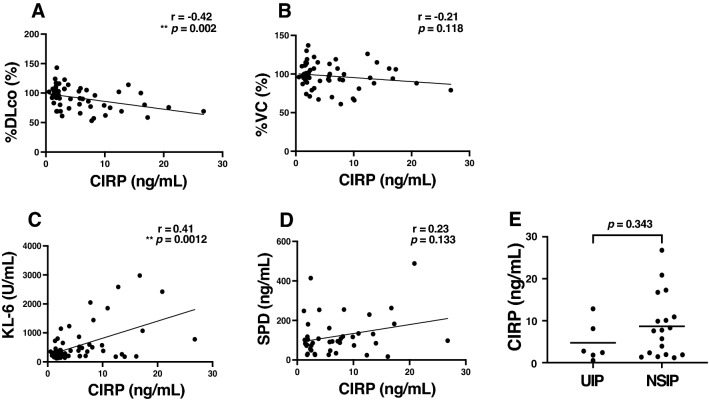
Correlation between serum CIRP levels and ILD parameters. (**A**,**B**) Negative correlation between serum cold-inducible RNA-binding protein (CIRP) levels and percent predicted diffusing capacity for carbon monoxide (%DL_CO_, A) or the percent predicted vital capacity (%VC, B) in systemic sclerosis (SSc) patients. (**C**,**D**) Positive correlation between serum CIRP levels and serum Krebs von den Lungen-6 (KL-6, **C**) or surfactant protein D (SP-D*,* **D**) in SSc patients. (**E**) Serum CIRP levels in SSc patients with usual interstitial pneumonia (UIP) and nonspecific interstitial pneumonia (NSIP). The solid lines represent the regression lines (**A**–**D**). **p < 0.01.

Serum CIRP levels negatively correlated with the percent predicted diffusing capacity for carbon monoxide (%DL_CO_) (*r* = − 0.42, *p* = 0.002; Fig. 2A), while they showed no significant correlation with the percent predicted vital capacity (%VC) (*r* = − 0.21, *p* = 0.118; Fig. 2B). We also investigated the correlation between serum CIRP levels and well-known serological markers of ILD such as serum KL-6 and SP-D^13^. Serum CIRP levels showed positive correlations with levels of KL-6 (*r* = 0.41, *p* = 0.0012; Fig. 2C). Though they showed no significant correlation with SP-D, they have similar trends (*r* = 0.23, *p* = 0.133; Fig. 2D). Considering the classification of ILD, the number of patients with usual interstitial pneumonia (UIP) and nonspecific interstitial pneumonia (NSIP) are 6 and 18, respectively, and there is no significant difference in the serum CIRP levels between them (2.58 [1.50–9.30] vs. 7.68 ng/mL [1.92–12.36], *p* = 0.343; Fig. 2E). Additionally, we performed a subgroup analysis comparing serum CIRP in ILD-positive and -negative patients in anti-topo I antibodies-positive or -negative patients. There is no significant difference in all subgroup analyses: patients with anti-topo I antibodies and without ILD vs. patients with anti-topo I antibodies and with ILD (5.87 [5.62–6.24] vs. 7.59 ng/mL [1.93–10.10], *p* = 0.753), patients without anti-topo I antibodies and without ILD vs. patients without anti-topo I antibodies and with ILD (2.14 [1.70–3.17] vs. 2.76 ng/mL [1.98–7.80], *p* = 0.677), patients with ILD and without anti-topo I antibodies vs. patients with ILD and with anti-topo I antibodies (2.76 [1.98–7.80] vs. 7.59 ng/mL [1.93–10.10], *p* = 0.680), and patients without ILD and without anti-topo I antibodies vs. patients without ILD and with anti-topo I antibodies (2.14 [1.70–3.17] vs. 5.87 ng/mL [5.62–6.24], *p* = 0.753). These data sets indicate that both ILD and anti-topo I might have a greater effect on serum CIRP levels. However, further analyses are desirable, as there are potential limitations due to the small sample size.

### Decrease in serum CIRP levels after treatment

**Figure 3:**
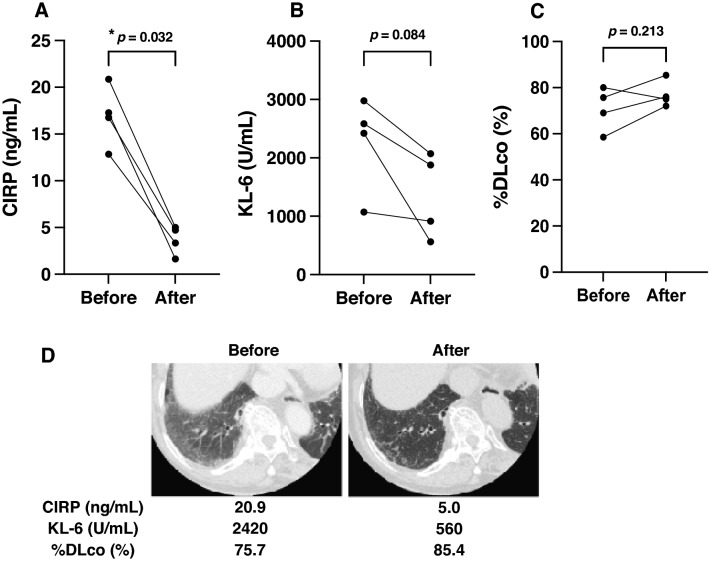
Decrease in serum CIRP levels of SSc patients after treatment for SSc-ILD. (**A**) Decrease in serum levels of cold-inducible RNA-binding protein (CIRP) in four patients with active systemic sclerosis (SSc) before (Before) and after (After) immunosuppressive treatments. (**B**) Decrease in serum levels of Krebs von den Lungen-6 (KL-6) in four patients with active SSc before (Before) and after (After) immunosuppressive treatments. (**C**) Changes in the percent predicted diffusing capacity for carbon monoxide (%DL_CO_) in four patients before (Before) and after (After) immunosuppressive treatments for SSc-ILD. (**D**) Images of chest computed tomography from a representative of four patients (**C**).

To explore the changes in serum CIRP levels after treatment, we included four SSc patients with both samples before and after treatment. They received corticosteroids or other immunosuppressants including i.v. cyclophosphamide (IVCY) pulses and tocilizumab. As a side note, tocilizumab was administered for coexisting rheumatoid arthritis and was expected to be effective for SSc-ILD, given that the US Food and Drug Administration approved tocilizumab for use in SSc-ILD. Serum CIRP levels significantly declined in patients with SSc after immunosuppressive therapies (*p* = 0.0312; Fig. 3A). As data to support the therapeutic effect, serum KL-6 levels before and after treatment in all 4 patients showed a downward trend (*p* = 0.084; Fig. 3B), although the difference was not statistically significant due to the small number of samples. Additionally, %DLco showed a decrease in one case, but an increase in the remaining three cases (*p* = 0.213; Fig. 3C). These four patients include a case whose radiological findings in chest computed tomography (CT) showed markedly reduced ground-glass opacities along with improving her ILD parameters after the treatment for SSc-ILD (Fig. 3D).

## Discussion

In this study, serum CIRP levels were significantly increased in dcSSc patients compared with lcSSc and healthy controls. From the perspective of antibodies, there was an association between serum CIRP levels and the presence of anti-topo I antibodies. Furthermore, serum CIRP levels positively correlated with the frequency and severity of ILD. Further analysis to determine whether anti-topo I antibodies or ILD had a greater effect on serum CIRP revealed that correlations between CIRP and KL-6 were observed regardless of the antibody type. Moreover, elevated serum CIRP levels in SSc patients with anti-topo I antibodies were associated with a high proportion of SSc-ILD patients in SSc patients with anti-topo I antibodies. These results suggest that CIRP may play a role in the development of SSc-ILD rather than specific pathogenesis in patients with anti-topo I antibodies.

This study has two limitations. First, we only conducted a single-center retrospective cohort study with a relatively small sample size of SSc patients, especially for subgroup analyses comparing CIRP levels between positive and negative cases of ILD and anti-topo I antibodies. More studies based on a larger cohort in additional sites are necessary to deepen our findings. Second, most serum CIRP levels were only measured once before the administration of treatment. Therefore, in terms of Fig. 3A, further samples are necessary to determine whether the serum levels of CIRP could be a useful monitoring tool to evaluate the effect of treatment for SSc-ILD.

Several recent studies reported the association between serum CIRP levels and autoinflammatory diseases such as rheumatoid arthritis, systemic lupus erythematosus, and adult-onset Still’s disease^14, 15^. To our knowledge, this is the first known study of serum CIRP levels in SSc. Recent studies have shown that CIRP binds the TLR4-MD2 complex, acting as a DAMP^16^. The expression of TLR4 is increased in the skin and lungs of patients with SSc and can heighten the sensitivity of fibroblasts to the profibrotic effects of TGFβ^17^. Mice lacking TLR4 had substantially less fibrosis and lower IL-6 levels than wild-type mice with bleomycin-induced fibrosis^18^. Moreover, a recent study has demonstrated that both TGFβ and IL-6 play an important role in fibrosis within SSc^16^. Our data revealed that increased serum CIRP levels were associated with ILD in SSc patients. Thus, CIRP may contribute to the development of SSc-ILD by stimulating TLR4-MD2. Further studies with genetically engineered mice are needed to prove and reveal the detailed mechanism.

In our study, serum CIRP levels showed positive correlations with KL-6. Though serum CIRP levels showed no significant correlation with SP-D, they have similar trends. It is generally reported that KL-6 is correlated with the progression of fibrosis and SP-D is correlated with the extent of alveolitis^19^. These results might suggest that CIRP shows a stronger correlation with the progression of fibrosis rather than the extent of alveolitis. However, it is likely that the difference in the result of KL-6 and SP-D is partly due to the small number of cases in this study and it is desirable to increase the number of cases in the future. Moreover, serum CIRP levels negatively correlated with %DL_CO_, while they showed no significant correlation with %VC. It has been reported that severe skin thickness of the chest wall in SSc patients potentially affects the pulmonary function test^20^. In our cases, %VC may be affected by the skin thickness of SSc patients. Therefore, serum CIRP levels might be more correlated with %DL_CO_ than with %VC. A previous study reported that serum KL-6 correlated with %DL_CO_ and could be a monitoring tool for SSc-ILD activity^21^. Given these literatures, our results suggest that CIRP might be useful in monitoring the severity of SSc-ILD^21^.

Additionally, serum CIRP levels significantly declined in SSc patients after treatment, along with decreased serum levels of KL-6 (Fig. 3B). These facts suggest that CIRP could be a more useful monitoring tool for SSc-ILD activity in monitoring the response of individual cases to a variety of treatments^22^. To analyze the difference in therapeutic effects between each treatment in detail, we would include more samples in the further study. In terms of therapy, a small molecule selectively targeting TLR4 signaling might provide new opportunities for preventing as well as reversing organ fibrosis in SSc and targeted therapy for SSc^23^. Given our results and the interaction between CIRP and TLR4-MD2 complex, we get a new hypothesis that a small molecule targeting CIRP signaling could be a crucial way of treating SSc.

In summary, here we showed that serum CIRP levels were increased in the serum of SSc patients. Moreover, increased serum CIRP levels were associated with greater frequency and severity of SSc-ILD, while not with other clinical manifestations of SSc. Although further studies are required to clarify the specific role of CIRP in the development of SSc-ILD, our results suggest that serum CIRP levels may serve as a useful serological marker of SSc-ILD in terms of disease activity and therapeutic effects. Our findings on CIRP hold the potential to pave the way for a novel design for the treatment of patients with SSc-ILD.

## Materials and methods

### SSc patients and controls subjects

SSc patients who came to the Department of Dermatology at The University of Tokyo were diagnosed on the basis of the 2013 ACR/EULAR classification criteria^24^. Serum samples, frozen at – 80 °C until assayed, were obtained from 60 SSc patients (56 women, four men; median, age [25–75th percentiles], 56.0 years[48.0–68.0]; disease duration, 2.0 years [1.0–7.0]) and 20 healthy controls (19 women, one man; age, 52.0 years [48.0–59.0]) after getting written informed consent according to the Declaration of Helsinki and institutional approval. The experimental protocols were approved by the Research Ethics Committee of the Faculty of Medicine of The University of Tokyo. This study was carried out in accordance with ethical guidelines and regulations. SSc patients treated with corticosteroids or other immunosuppressants prior to their first visits were excluded. Healthy controls had no underlying disease and serums were collected at the time of benign tumour resection in the Department of Dermatology at The University of Tokyo Hospital. SSc patients were categorized by LeRoy’s classification system^25^: 30 diffuse cutaneous SSc (dcSSc) patients (age, 57.0 years [45.7–71.5]; disease duration, 2.0 years [1.0–8.0]) and 30 limited cutaneous SSc (lcSSc) patients (age, 56.0 years [51.5–61.5]; disease duration, 3.0 years [0.5–6.8]). A diagnosis and classification of ILD were confirmed by high-resolution CT and supported by clinical and physiological findings, based on the previously reported SSc guidelines including SSc-ILD^26, 27^. For four SSc patients, pre- and post-treatment time series serums were available, and both of them were measured.

### Measurement of serum CIRP levels

Serum CIRP levels were examined using enzyme-linked immunosorbent assay kits (MBL, Nagoya, Japan; Code No, CY-8103). Briefly, polystyrene plates coated with anti-CIRP antibodies were incubated with fourfold diluted serum at room temperature for 1 h. The wells were washed and incubated at room temperature for 1 h with horseradish peroxidase-conjugated anti-CIRP antibodies. The wells were washed again, supplemented with tetramethylbenzidine, and incubated at room temperature for 10 min. Finally, sulfuric acid was added to terminate the reaction. The absorbance at 450 nm was measured by MULTISKAN FC^®^ (Thermo Fisher SCIENTIFIC). Serum CIRP levels were calculated from a standard curve.

### Measurement of serum levels of KL-6, SP-D, and CRP

Krebs von den Lungen-6 (KL-6) and surfactant protein D (SP-D) are useful serum markers for ILD. Serum levels of KL-6 and SP-D were determined with enzyme-linked immunosorbent assay kits (Eisai, Tokyo, Japan, and Yamasa, Chiba, Japan, respectively)^28^. The cut-off values for these antigens were set at 500 U/mL for KL-6 and 110 ng/mL for SP-D, respectively. C-reactive protein (CRP), an acute-phase reactant, has been utilized as a marker of infection and inflammation^16^, and higher baseline CRP levels in SSc have been reported to be associated with SSc-ILD^29^. Then, CRP serum levels were also determined by the latex agglutination nephelometric immunoassay test as described previously^28^. The cut-off value for CRP was set at 3.00 mg/L.

### Clinical assessments

The clinical data of SSc patients were collected by a retrospective review of medical records. Disease onset was defined as the first clinical event that was a clear manifestation of SSc other than Raynaud’s phenomenon. Disease duration was defined as the term between the onset and the time serum was obtained. The modified Rodnan total skin thickness score was used for evaluating the extent and severity of skin thickness^30^. The details of the assessment for other organ involvement are described in Table 1.

### Statistical analysis

Prism (GraphPad, ver. 9) software was used for all statistical analyses. Statistical analysis was performed by two ordinary one-way analysis of variance followed by Tukey’s post-hoc test for frequency analysis and Pearson’s rank correlation coefficient for clinical correlations. Two-tailed, unpaired Mann–Whitney *U* tests or paired *t*-tests were performed when comparing only two groups. A value of *P* < 0.05 was considered statistically significant. In graphs, horizontal bars represent the median of each group. Lines in correlation plots represent the regression lines for the relationship between two variables.

## Data Availability

The original data analyzed in this study are available from the corresponding author upon reasonable request.

## References

[1] Asano Y (2018). Systemic sclerosis. J. Dermatol..

[2] Tyndall AJ (2010). Causes and risk factors for death in systemic sclerosis: A study from the EULAR Scleroderma Trials and Research (EUSTAR) database. Ann. Rheum. Dis..

[3] Elhai M (2019). Performance of candidate serum biomarkers for systemic sclerosis-associated interstitial lung disease. Arthritis Rheumatol..

[4] Aden N (2010). Epithelial cells promote fibroblast activation via IL-1α in systemic sclerosis. J. Investig. Dermatol..

[5] Nikitorowicz-Buniak J, Shiwen X, Denton CP, Abraham D, Stratton R (2014). Abnormally differentiating keratinocytes in the epidermis of systemic sclerosis patients show enhanced secretion of CCN2 and S100A9. J. Investig. Dermatol..

[6] Ellson CD, Dunmore R, Hogaboam CM, Sleeman MA, Murray LA (2014). Danger-associated molecular patterns and danger signals in idiopathic pulmonary fibrosis. Am. J. Respir. Cell Mol. Biol..

[7] Qiang X (2013). Cold-inducible RNA-binding protein (CIRP) triggers inflammatory responses in hemorrhagic shock and sepsis. Nat. Med..

[8] Nishiyama H (1997). Cloning and characterization of human CIRP (cold-inducible RNA-binding protein) cDNA and chromosomal assignment of the gene. Gene.

[9] Aziz M, Brenner M, Wang P (2019). Extracellular CIRP (eCIRP) and inflammation. J. Leukoc. Biol..

[10] Zhong P, Huang H (2017). Recent progress in the research of cold-inducible RNA-binding protein. Future Sci. OA.

[11] Bazid H (2022). Expression of cold-inducible RNA binding protein in psoriasis. J. Immunoassay Immunochem..

[12] Hozumi H (2021). Clinical significance of cold-inducible RNA-binding protein in idiopathic pulmonary fibrosis. Chest.

[13] Yanaba K, Hasegawa M, Takehara K, Sato S (2004). Comparative study of serum surfactant protein-D and KL-6 concentrations in patients with systemic sclerosis as markers for monitoring the activity of pulmonary fibrosis. J. Rheumatol..

[14] Fujita Y (2021). Clinical relevance for circulating cold-inducible RNA-binding protein (CIRP) in patients with adult-onset Still’s disease. PLoS ONE.

[15] Yoo IS (2018). Serum and synovial fluid concentrations of cold-inducible RNA-binding protein in patients with rheumatoid arthritis. Int. J. Rheum. Dis..

[16] Lujan DA, Ochoa JL, Hartley RS (2018). Cold-inducible RNA binding protein in cancer and inflammation. Wiley Interdiscip. Rev. RNA.

[17] Bhattacharyya S (2013). Toll-like receptor 4 signaling augments transforming growth factor-β responses: A novel mechanism for maintaining and amplifying fibrosis in scleroderma. Am. J. Pathol..

[18] Takahashi T (2015). Amelioration of tissue fibrosis by toll-like receptor 4 knockout in murine models of systemic sclerosis. Arthritis Rheumatol..

[19] Takahashi H (2000). Serum surfactant proteins A and D as prognostic factors in idiopathic pulmonary fibrosis and their relationship to disease extent. Am. J. Respir. Crit. Care Med..

[20] Nawata T, Shirai Y, Suzuki M, Kuwana M (2021). Chest wall muscle atrophy as a contributory factor for forced vital capacity decline in systemic sclerosis-associated interstitial lung disease. Rheumatology.

[21] Yamakawa H (2017). Serum KL-6 and surfactant protein-D as monitoring and predictive markers of interstitial lung disease in patients with systemic sclerosis and mixed connective tissue disease. J. Thorac. Dis..

[22] Sumida H (2014). Successful experience of rituximab therapy for systemic sclerosis-associated interstitial lung disease with concomitant systemic lupus erythematosus. J. Dermatol..

[23] Bhattacharyya S (2018). Pharmacological inhibition of tolllike receptor-4 signaling by TAK242 prevents and induces regression of experimental organ fibrosis. Front. Immunol..

[24] van den Hoogen F (2013). 2013 classification criteria for systemic sclerosis: An American college of rheumatology/European league against rheumatism collaborative initiative. Ann. Rheum. Dis..

[25] LeRoy EC (1988). Scleroderma (systemic sclerosis): Classification, subsets and pathogenesis. J. Rheumatol..

[26] Asano Y (2018). Diagnostic criteria, severity classification and guidelines of systemic sclerosis. J. Dermatol..

[27] Solomon JJ (2013). Scleroderma lung disease. Eur. Respir. Rev..

[28] Sumida H (2018). Prediction of therapeutic response before and during i.v. cyclophosphamide pulse therapy for interstitial lung disease in systemic sclerosis: A longitudinal observational study. J. Dermatol..

[29] Liu X (2013). Does C-reactive protein predict the long-term progression of interstitial lung disease and survival in patients with early systemic sclerosis?. Arthritis Care Res..

[30] Clements P (1995). Inter and intraobserver variability of total skin thickness score (modified Rodnan TSS) in systemic sclerosis. J. Rheumatol..

